# The peripheral blood transcriptome in septic cardiomyopathy: an observational, pilot study

**DOI:** 10.1186/s40635-019-0271-0

**Published:** 2019-10-24

**Authors:** Meghan M. Cirulis, Sarah J. Beesley, Emily L. Wilson, Chris Stubben, Troy D. Olsen, Eliotte L. Hirshberg, Lane M. Smith, Michael J. Lanspa, Theodore P. Abraham, Colin K. Grissom, Matthew T. Rondina, Samuel M. Brown

**Affiliations:** 1 Division of Respiratory, Critical Care, and Occupational Pulmonary Medicine, Department of Medicine, University of Utah, Salt Lake City, UT USA; 2 Pulmonary and Critical Care Division, Department of Medicine, Intermountain Medical Center, Shock Trauma Intensive Care Unit, 5121 South Cottonwood Street, Murray, UT 84107 42 USA; 3 Critical Care Echocardiography Service, Intermountain Medical Center, Murray, UT USA; 4 Bioinformatics Shared Resource, Huntsman Cancer Institute, University of Utah, Salt Lake City, UT USA; 5 Department of Emergency Medicine, Wake Forest School of Medicine, Winston Salem, NC USA; 6 Division of Cardiology, Department of Medicine, UCSF, San Francisco, CA USA; 7 Molecular Medicine Program, University of Utah, Salt Lake City, UT USA

**Keywords:** Global longitudinal strain, Interferon, Sepsis

## Abstract

**Background:**

Septic cardiomyopathy (SCM) is common in sepsis and associated with increased morbidity and mortality. Left ventricular global longitudinal strain (LV GLS), measured by speckle tracking echocardiography, allows improved identification of impaired cardiac contractility. The peripheral blood transcriptome may be an important window into SCM pathophysiology. We therefore studied the peripheral blood transcriptome and LV GLS in a prospective cohort of patients with sepsis.

**Results:**

In this single-center observational pilot study, we enrolled adult patients (age > 18) with sepsis within 48 h of admission to the ICU. SCM was defined as LV GLS > − 17% based on echocardiograms performed within 72 h of admission. We enrolled 27 patients, 24 of whom had high-quality RNA results; 18 (75%) of 24 had SCM. The group was 50% female and had a median (IQR) age of 59.5 (48.5–67.0) years and admission APACHE II score of 21.0 (16.0–32.3). Forty-six percent had septic shock. After filtering for low-expression and non-coding genes, 15,418 protein coding genes were expressed and 73 had significantly different expression between patients with vs. without SCM. In patients with SCM, 43 genes were upregulated and 30 were downregulated. Pathway analysis identified enrichment in type 1 interferon signaling (adjusted *p* < 10^−5^).

**Conclusions:**

In this hypothesis-generating study, SCM was associated with upregulation of genes in the type 1 interferon signaling pathway. Interferons are cytokines that stimulate the innate and adaptive immune response and are implicated in the early proinflammatory and delayed immunosuppression phases of sepsis. While type 1 interferons have not been implicated previously in SCM, interferon therapy (for viral hepatitis and Kaposi sarcoma) has been associated with reversible cardiomyopathy, perhaps suggesting a role for interferon signaling in SCM.

## Background

Current consensus defines sepsis as life-threatening organ dysfunction in the setting of an abnormal host response to infection. Cardiac dysfunction in sepsis, i.e., “septic cardiomyopathy (SCM),” is common and if severe, may contribute to the dysfunction of other organs in the septic patient [1]. Left ventricular global longitudinal strain (LV GLS) has been identified as an accurate, non-invasive measure of cardiac contractility, allowing improved interrogation of the pathophysiology of SCM [2–7]. Compared to the traditional measurement of LV ejection fraction (EF), LV GLS is less dependent on cardiac preload and afterload—conditions that can change often and rapidly during the course of sepsis and septic shock [3, 4]. Mechanistically, numerous factors related to the dysregulated inflammatory response have been hypothesized to play a role in the development of SCM, although none are confirmed to be causative [8–10].

Peripheral blood transcriptome analysis enables exploration of gene expression profiles in disease states of interest. No study has examined the peripheral blood transcriptome in patients with SCM. In this pilot study, we compared the peripheral blood transcriptional profile of septic patients with and without SCM as defined by LV GLS.

## Methods

### Study design and patient data

**Fig. 1:**
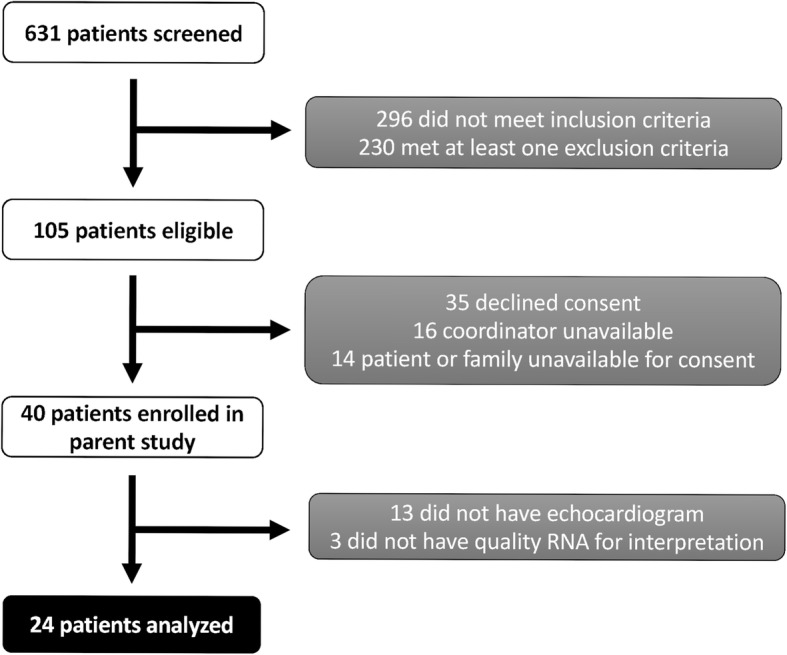
Consort diagram illustrating patient selection for parent and sub-study

We report a sub-study of a larger prospective single-center observational study of post-sepsis cognitive impairment (NCT03015584) (Fig. 1). Full parent study eligibility criteria are reported on the ClinicalTrials.gov website; briefly, we enrolled patients with sepsis (SEPSIS-3 criteria) admitted to a study ICU from June 2015 to July 2017. Patients had to be enrolled within 48 h of admission to the study ICU and were excluded for the following: onset of sepsis or septic shock > 24 h after hospital admission or transfer to the ICU was > 48 h after admission (with admitting diagnosis of sepsis); transfer from another hospital except directly from emergency room; prior prespecified neurologic or psychiatric comorbidities or cardiac surgery; “Do Not Resuscitate/Do not Intubate” order prior to study enrollment; known pregnancy; primary diagnosis of drug overdose; or attending physician deemed aggressive care unsuitable or the patient was not expected to survive 48 h. Patients with a clinical echocardiogram within the first 72 h of admission were included in this sub-study. We collected blood for whole blood RNA sequencing at time of enrollment. We also gathered patient demographics and severity of illness parameters.

### Laboratory analysis

RNA sequencing was performed on blood drawn at the time of enrollment using established methods (Illumina platform). Blood was drawn in PAXgene tubes and immediately frozen at − 20 °C. We extracted RNA from peripheral whole blood using PAXgene Blood RNA Kit IVD (Qiagen Cat #762164), with ~ 1mcg of RNA isolated from whole blood. One hundred nanograms of this RNA was used for Next Generation RNA sequencing (using the Illumina kits with Ribo-Zero Globin processing, 50 cycle single read), aligned to H_sapiens_Feb_2009_B37. Total RNA samples (100–500 ng) were hybridized with Ribo-Zero Globin to substantially deplete both globin RNA and rRNA species from the samples. Stranded RNA sequencing libraries were prepared as described using the Illumina TruSeq Stranded Total RNA Kit with Ribo-Zero Globin (RS-122-2501 and RS-122-2502). Purified libraries were qualified on an Agilent Technologies 2200 TapeStation using a D1000 ScreenTape assay (cat# 5067-5582 and 5067-5583). The molarity of adapter-modified molecules was defined by quantitative PCR using the Kapa Biosystems Kapa Library Quant Kit (cat#KK4824). Individual libraries were normalized to 10 nM, and equal volumes were pooled in preparation for Illumina sequence analysis. Sequencing libraries (25 pM) were chemically denatured and applied to an Illumina HiSeq v4 single-read flow cell using an Illumina cBot. Hybridized molecules were clonally amplified and annealed to sequencing primers with reagents from an Illumina HiSeq SR Cluster Kit v4-cBot (GD-401-4001). Following transfer of the flowcell to an Illumina HiSeq 2500 instrument (HCSv2.2.38 and RTA v1.18.61), a 50-cycle single-read sequence run was performed using HiSeq SBS Kit v4 sequencing reagents (FC-401-4002).

The human GRCh38 FASTA and gene transfer format (GTF) files were downloaded from Ensembl release 87, and the reference database was created using STAR version 2.5.2b [11] with splice junctions optimized for 50 base pair reads. Reads were trimmed of adapters and aligned to the reference database using STAR in two-pass mode to output a Binary Alignment Map (BAM) file sorted by coordinates. Mapped reads were assigned to annotated genes in the GTF file using featureCounts version 1.5.1 [12]. The output files from FastQC, Picard CollectRnaSeqMetrics, STAR, and featureCounts were summarized using MultiQC [13] to check for any sample outliers.

### Statistical analysis

Differentially expressed genes were identified using a 10% false discovery rate (FDR) with DESeq2 version 1.18 [14]. All the expressed genes were sorted by log_2_ fold change and compared to Reactome pathways using gene set enrichment analysis (GSEA) [15]. In addition, significant genes were compared to the same pathways using Fisher’s exact test to find significant overlaps. We performed analyses in R version 3.2.3 (Vienna, Austria) [16].

### Echocardiography

We used clinically acquired two-dimensional echocardiograms obtained within 72 h of admission performed at the discretion of the treating physician. In the study ICU, clinical echocardiograms are routinely obtained for the management of patients with serious sepsis and septic shock. We retrospectively measured LV GLS following a standard protocol on apical four-chamber views using the Image-Arena platform (Tomtec Imaging Systems, Unterschleissheim, Germany) as we have applied in other cohorts [3]. We used the best single cardiac cycle and rejected images if we could not perform tracking on two or more adjacent segments. We defined abnormal strain as greater than − 17%, consistent with prior work in patients with septic shock [17, 18].

## Results

### Patient characteristics

A total of 631 patients were screened for the parent study, 40 of whom were enrolled. Of those 40 patients, 27 patients had an echocardiogram performed in the first 72 h and 24 had RNA results of sufficient quality for interpretation (Fig. 1). Median time to first echocardiogram was 1.3 h (IQR 0.3–3.8) from ICU admission; median time between RNA sample collection and echocardiogram was 14.5 h (IQR 10.7–21.4). Characteristics of the cohort are listed in Table 1, including source of sepsis when available. Eighteen of the 24 patients (75%) met LV GLS criteria for SCM. Overall, the group was 50% female and had a median (IQR) age of 59.5 years (48.5–67.0) and admission APACHE II score of 21.0 (16.0–32.3). Forty-six percent of the cohort met the SEPSIS-3 criteria for septic shock; a similar rate of septic shock was seen between those with and without SCM (44% vs. 50%). Median LV ejection fraction was 57% in the SCM group and 66% in patients without SCM. Those with SCM had more comorbid medical conditions than those without (median Elixhauser Comorbidity Index 17.0 vs. 9.5). ICU length of stay was 3.7 days vs. 2.9 days in those with vs. those without SCM, and mortality was 22% vs. 0% in the two groups. Source of infection was identified in 94% of those with SCM and 67% of those without; pneumonia was the most frequent type of infection in the patients with SCM (39%). The rate of infection with gram-positive and gram-negative organisms was similar between the two groups. Only two patients were identified to have a viral infection (one isolated, one concurrent with a bacterial infection), both of which were in the SCM group.

**Table 1:** Characteristics of patients with and without septic cardiomyopathy

	Septic cardiomyopathy (*n* = 18)	No septic cardiomyopathy (*n* = 6)
Age (years); median (IQR)	62.0 (43.8–67.0)	57 (54.0–64.5)
Admission APACHE II (points); median (IQR)	18.5 (14.5–36.0)	22.5 (20.5–23.0)
Female, *n* (%)	7 (39%)	5 (83%)
Septic shock, *n* (%)	8 (44%)	3 (50%)
Elixhauser comorbidity score (points); median (IQR)	17 (6.8–26.0)	9.5 (4.3–11.8)
Mechanically ventilated, *n* (%)	7 (39%)	1 (17%)
Duration of mechanical ventilation among those ventilated (days); median (IQR)	1.7 (0.8–2.4)	2.8^a^
ICU length of stay; median (IQR)	3.7 (2.5–4.7)	2.9 (2.5–3.3)
Hospital length of stay; median (IQR)	8.0 (5.0–13.6)	5.6 (5.1–6.0)
90-day mortality	4 (22%)	0 (0%)
Echocardiogram data Median (IQR)		
LV GLS	− 11.4 (− 13.6 to − 8.4)	−19.8 (− 21.0 to − 17.3)
LV ejection fraction %	57 (45–66)	66 (64–68)
Source of infection, *n* (%)		
Pneumonia	7 (39%)	1 (17%)
Urinary tract infection	3 (17%)	1 (17%)
Skin and soft tissue	4 (22%)	1 (17%)
Intra-abdominal	2 (11%)	1 (17%)
CLABSI	1 (6%)	0 (0%)
Unknown	1 (6%)	2 (33%)
Bacteremia present	7 (39%)	2 (33%)
Organism type, *n* (%)		
Bacterial	13 (72%)	3 (50%)
Viral	1 (6%)	0
Mixed^b^	1 (6%)	0
Unknown	3 (17%)	3 (50%)
Microbiology^c^, *n* (%)		
Gram positive	10 (55%)	3 (50%)
Gram negative	6 (33%)	2 (33%)

*LV* left venticular, *CLABSI* central line-associated bloodstream infection

^a^Only one patient mechanically ventilated and duration was 2.8 days

^b^Bacterial and viral infection

^c^Some subjects had polymicrobial infection (gram positive and gram negative)

### Transcriptional profiling

**Fig. 2:**
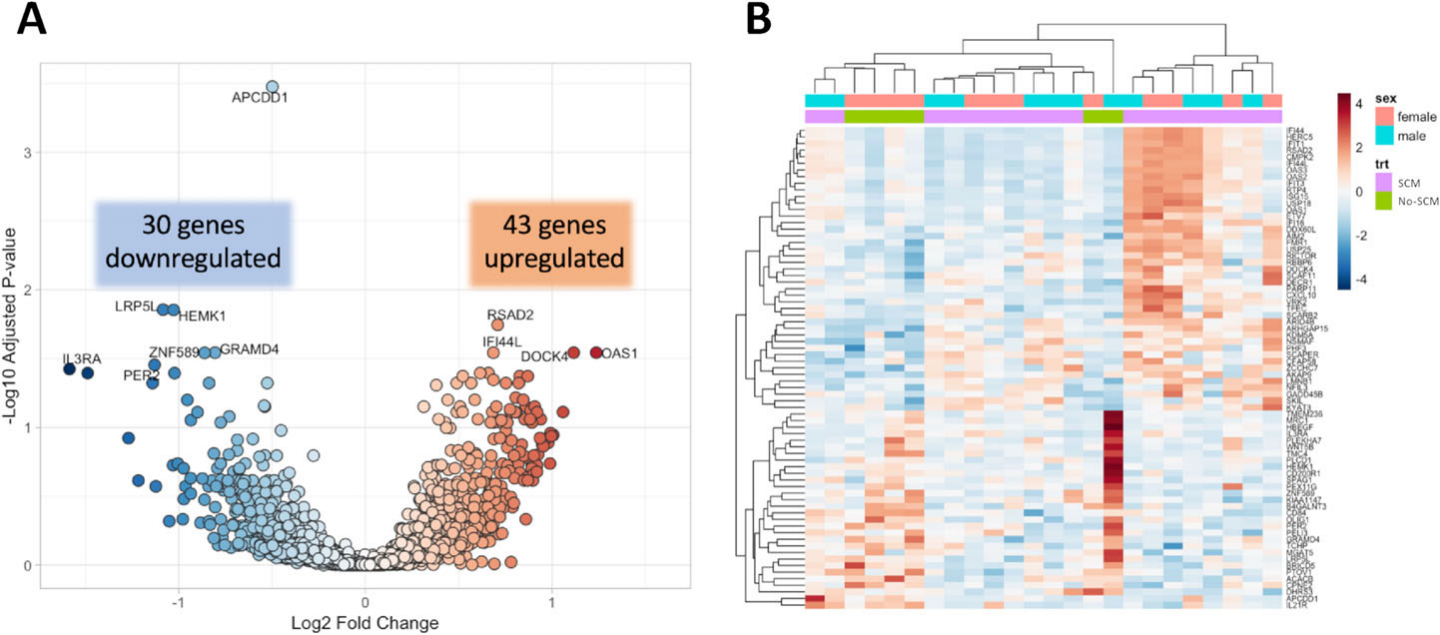
RNAseq analysis of patients with septic cardiomyopathy compared to those without SCM demonstrates differential gene expression. **a** Volcano plot illustrating fold differences in gene expression in septic cardiomyopathy vs. non-septic cardiomyopathy subjects. **b** Heatmap of differentially expressed mRNAs using unsupervised hierarchical clustering. SCM, septic cardiomyopathy

After filtering for low-expression and non-coding genes, 15,418 protein coding genes were identified, and 73 had significantly different expression between patients with vs. without SCM. In the patients with SCM, 43 genes were upregulated and 30 were downregulated, as shown in the volcano plot (Fig. 2a). Unsupervised hierarchical clustering of the differentially expressed mRNA demonstrates the patterns of expression within the SCM and non-SCM groups displayed in Fig. 2b.

Pathway analysis using GSEA identified significant enrichment in several pathways; the most enriched pathways (positive or negative) are displayed in Table 2. Notably, we observed positive enrichment in interferon (IFN) signaling (adjusted *p* < 0.005) in patients with SCM; the top pathway in terms of differential expression was type 1 IFN signaling.

**Table 2:** Top pathways in gene sequence enrichment analysis (GSEA)

Top pathways in gene sequence enrichment analysis (GSEA)	Normalized enrichment score	Direction of enrichment
Type 1 interferon signaling	2.96	↑
Interferon signaling	2.68	↑
Peptide chain elongation	2.51	↑
Interferon gamma signaling	2.42	↑
RNA Pol I promoter opening	− 2.80	↓
Telomere maintenance	− 2.70	↓
Packaging of telomere ends	− 2.66	↓
RNA Pol I transcription	− 2.65	↓

Adjusted *p* < 0.05 for all pathways

**Fig. 3:**
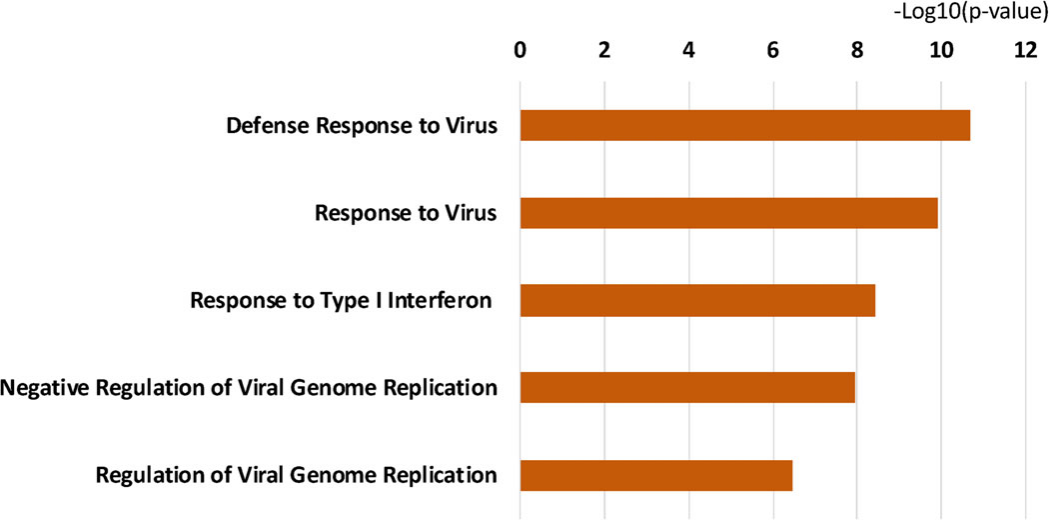
Gene ontology category overrepresentation for dysregulated genes in patients with septic cardiomyopathy

Similarly, type 1 IFN signaling was also the top pathway in overrepresentation pathway analysis (*p* < 10^−7^). Figure 3 demonstrates the gene ontology category overrepresentation for genes dysregulated in patients with SCM. The most significantly overrepresented categories were those related to viral response to infection and type 1 IFN signaling. We attempted an exploration of the relationship between infecting microorganism and the transcriptome, but the small number of individuals in each group and the presence of polymicrobial infection in a substantial proportion prevented a successful analysis.

## Discussion

In this exploratory pilot study, SCM (as defined by LV GLS of > − 17%) was associated with upregulation of genes in IFN signaling pathways, including type 1 IFN and IFN gamma. Repressed pathways include those important for RNA polymerase function and telomere maintenance and packaging. Type 1 IFN signaling was the top pathway in both GSEA and overrepresentation pathway analysis.

Type 1 IFNs (especially α and β) are widely expressed and signal through a specific receptor (IFNAR) to induce changes in the expression of hundreds of downstream genes [19]. Ligation of intracellular (viral) or cell surface (bacterial) pattern recognition receptors (PRR) leads to activation of nuclear transcription factors that drive expression of type 1 IFNs [19]. Type 1 IFN signaling was initially recognized for antiviral properties, but studies in recent decades have identified the pleotropic effects of type 1 IFN signaling in a variety of bacterial infections as well [19]. IFN α and β have been studied in sepsis, mostly in gram-negative endotoxemia, and are implicated in both the early proinflammatory and delayed immunosuppressive phases [20].

Although IFN signaling has not been directly implicated in prior investigations of the pathogenesis of SCM, there is substantial overlap between factors associated with cardiac dysfunction in sepsis and the canonical IFN pathway. For instance, activation of toll-like receptor 4 (TLR4) by lipopolysaccharide (LPS) in gram-negative infection signals through intracellular TIR-domain-containing adaptor-inducing beta interferon (TRIF) to increase expression of IFN-β. TLR4 activation also signals through MyD88 to activate the NFκB transcription factor which increases expression of classic inflammatory cytokines IL-1 and TNFα [20]. There is significant cross talk between TNFα/IL-1 and type 1 IFN signaling pathways, mostly in a counterregulatory fashion, but TNFα has also been shown to stimulate production of IFN-β in an autocrine signaling loop [21–23]. TLR4, IL-1, and TNFα have all been hypothesized to play a role in the development of SCM [24–27]. Additionally, acting through the STAT transcription factor, IFN-β increases expression of inducible nitric oxide synthase (iNOS) and thus nitric oxide (NO) production. IFN gamma may also interact with LPS in gram-negative sepsis to induce NOS activity [28]. It has recently been suggested that endothelial NO overproduction may lead to both early and late cardiac dysfunction in sepsis [29–31].

Adding to the biologic plausibility that type 1 IFN signaling may play a role in the development of SCM is the historical experience with IFN chemotherapy in select cancers (e.g., Kaposi sarcoma) and viral hepatitis. A review of 44 patients with interferon-related cardiotoxicity identified five cases of frank cardiomyopathy. Other reported effects included arrhythmia, myocardial ischemia, and sudden death. In the majority of cases, IFN-associated cardiac dysfunction reversed with drug discontinuation [32]. Occasionally, IFN-associated cardiac function may be irreversible and lead to death. In one report, autopsy in such a case revealed drug-induced cardiomyopathy [33]. The mechanism by which IFN α causes cardiac toxicity is currently unknown.

Our study has several limitations. First, sample size was small, and most patients had SCM, with an imbalance by sex between patients with and without SCM. These distributions, probably by chance, may limit confidence in results, although the imbalance by sex may also represent sex differences in cardiac function as observed in other disease states [34]. The causative organism was undetermined in 17% and 50% of subjects with and without SCM, respectively. Unlike standard testing for bacterial organisms (i.e., blood cultures), viral screening is not routine and therefore viral infections could be missed if not clinically suspected and tested for by the treating physician. Viral organisms are a classic trigger for the type 1 interferon pathway [19], and correlation with SCM may become apparent if all viral infections could be identified.

Another potential limitation of this study is the threshold of LV GLS chosen to define SCM. While we used a previously published definition of LV dysfunction (> − 17%) [17, 18], this limit may overestimate the frequency of SCM, as 75% of our study population met this criterion. The median LVEF in the SCM cohort was 57%, which is still considered normal by the American Society of Echocardiography guidelines [35]. The clinical relevance of mild LV dysfunction in the setting of sepsis and septic shock is unclear. A second possible explanation for the high prevalence of SCM in this study is selection bias. Subjects had to have a clinically acquired echocardiogram to be included in the study population, and treating clinicians at the study ICU are more likely to order an echocardiogram if a patient is hemodynamically unstable. This tendency may have led to exclusion of patients with milder forms of sepsis and inclusion of a higher proportion of patients with septic shock (46% overall), limiting the generalizability of the findings to a general sepsis cohort.

As is true for most studies of sepsis, patients may have been enrolled at various time points in the course of their sepsis [36]. Similarly, echocardiograms were performed at various points in the early phase of the ICU stay. Given that the natural history of SCM is at present undefined, the chosen time point (early vs. late sepsis) could alter the reported prevalence and the transcriptome results; late SCM may be associated with a different profile. Lastly, the transcriptomic analysis was not confirmed to correlate with protein expression of the molecules of interest in analyses of peripheral blood protein/cytokine levels. Future studies that control for time of sepsis onset and validate with proteomic analysis will improve the generalizability of transcriptome analyses.

## Conclusions

In this exploratory study of sepsis patients, canonical type 1 IFN signaling is upregulated in peripheral leucocytes among patients with SCM compared to those without SCM. Interferons are previously implicated in sepsis pathophysiology but have not to our knowledge been reported as a potential contributor to SCM. Multiple lines of evidence suggest possible biological plausibility, but further studies are required to determine whether IFN may in fact play an etiologic role in SCM.

## Data Availability

In order to protect patient privacy and comply with relevant regulations, identified data are unavailable. Requests for deidentified data from qualified researchers with appropriate ethics board approvals and relevant data use agreements will be processed by the Intermountain Office of Research, officeofresearch@imail.org.

## Abbreviations

GSEA: Gene set enrichment analysis
IFN: Interferon
LPS: Lipopolysaccharide
LV GLS: Left ventricular global longitudinal strain
SCM: Septic cardiomyopathy
TLR: Toll-like receptor
